# Survey of Notified Bodies reveals very limited use of conditional certification for high-risk medical devices

**DOI:** 10.3389/fmedt.2025.1504294

**Published:** 2025-02-03

**Authors:** A. Dobrzynska, J. C. Rejon-Parrilla, D. Epstein, J. Aranda-López, A. G. Fraser, J. A. Blasco-Amaro

**Affiliations:** ^1^ Health Technology Assessment Area (AETSA), Andalusian Public Foundation Progress and Health (FPS), Seville, Spain; ^2^ Department of Applied Economics, University of Granada, Granada, Spain; ^3^ Department of Cardiology, University Hospital of Wales, Cardiff, United Kingdom

**Keywords:** certificates of conformity, conditional approval, high-risk medical devices, Notified Bodies, Medical Device Regulation 2017/745

## Abstract

The aim was to identify the experiences of Notified Bodies (NBs) in Europe in applying restrictions or limitations to certificates for high-risk medical devices. A survey examining NB practices regarding restrictions or limitations applied to Class III and IIb implantable medical devices was conducted as part of the CORE-MD Horizon 2020 project. Thirteen NBs responded; three had issued certificates of conformity with restrictions or limitations. NBs reported challenges in collecting and providing data on conditional certification, which would likely increase their workload. Enhancing clarity of regulatory standards, improving data transparency, fostering stakeholders' collaboration, and providing targeted training are essential to ensure uniform and homogeneous application of conditional certifications across the EU.

## Introduction

The implementation of the European Union Medical Device Regulation (MDR) 2017/745 and the *in vitro* Diagnostic Medical Devices Regulation (IVDR) 2017/746 has marked a significant shift in the regulatory landscape for medical devices in the EU (1). These regulations have replaced the previous Medical Device Directive (MDD) and the Active Implantable Medical Device Directive (AIMDD) (93/42/EC and 90/385/EEC). One important aim has been to enhance the safety and effectiveness of medical devices. Key changes introduced by the MDR affected product classification and safety, clinical evaluation and investigations, and post-market clinical follow-up (PMCF). The MDR requires more robust clinical evidence for safety and performance, particularly for high-risk devices, and reclassifies some devices into higher-risk groups. Additionally, the MDR implemented the Unique Device Identification (UDI) system to improve traceability and strengthen post-market surveillance (2, 3). Stricter accreditation and enhanced oversight of Notified Bodies further ensure comprehensive conformity assessments, effectively addressing gaps in the previous MDD framework.

Medical devices include a wide range of products such as instruments, apparatus, appliances, materials, and articles used independently or in combination for healthcare applications (MDR, Article 2.1) (1). High-risk medical devices (class III and class IIb implantable; Article 51, MDR) encompass more complex and potentially life-preserving technologies, such as implantable cardiac devices and advanced surgical instruments (4).

Within the EU regulatory system, the Notified Bodies (NBs) have a central role. They are autonomous third-party organizations designated by EU Member States to assess and verify if medical devices conform with regulatory standards (5, 6). Under the MDD, NBs evaluated applications for approval of high-risk medical devices, in a procedure known as conformity assessment; if the NB judges a device to comply with EU safety and performance standards, it issues a Certificate of Conformity, enabling the manufacturer then to display a CE (Conformité Européenne) mark. With the introduction of the MDR, that basic role has not changed, but the level of clinical evidence required to place medical devices on the market has been increased by the MDR (MDR, Article 61; Annex II, Annex XIV) (1). NBs now have increased responsibilities, including enhanced assessment of the clinical data and of the updates of clinical evaluation from post-market surveillance, to ensure that high-risk devices maintain their safety and performance standards (3, 7). The workload of NBs has increased, so they have needed to increase personnel and organizational capacity to ensure that they comply with the new regulations (8, 9).

It is important from a clinical perspective that any regulatory system for medical devices includes options that will allow a limited number of devices to be approved in special circumstances – such as innovative devices that can be used for genuine unmet clinical needs, devices to be used in children, and devices required to treat orphan diseases or for rare indications.

Clinical evidence for new high-risk medical devices is often limited at the time of the first regulatory decision, in which case it may be possible to collect evidence after approval, to guide decisions throughout a product's lifecycle. In many countries this can be demanded as a condition of the initial approval. For example, in the United States and China, regulatory pathways describe a “conditional approval” procedure for certain high-risk medical devices (10, 11). In particular, the FDA's Breakthrough Devices Program facilitates conditional approval (12) allowing devices to enter the market based on somewhat immature evidence of safety and effectiveness, often in recognition of the urgency of the medical need for the device under evaluation, but with the requirement of ongoing post-market studies to mitigate such uncertainties. Similarly, China's National Medical Products Administration (NMPA), as outlined in its Guidelines for Conditional Approval for Marketing of Medical Devices, can permit market entry based on preliminary data for devices that address significant public health needs, contingent upon further post-market data collection and rigorous monitoring (13, 14). In Europe, for medicines, the European Medicines Agency (EMA) has an established route to grant conditional marketing authorizations on the basis of less comprehensive data than usually requested, when the medicine addresses an unmet medical need (15).

The EU Medical Device regulatory framework has never provided a dedicated pathway for “conditional certification” of medical devices. Under the MDD, however, NBs had authority to issue certificates of conformity with restrictions or limitations. They could require additional tests or evidence (Annex II point 4.3) (4). A Medical Device Guidance (MEDDEV) document from the European Commission addressed the use of devices for unmet medical need or as breakthrough products, suggesting that notified bodies might need to review more post-market data (MEDDEV 2.7/1 rev 4 section appendix A8. Article 4.8). With the introduction of the MDR, these provisions have been clarified and enhanced. The MDR now refers specifically to the need for limitations or restrictions in certification, when data are limited but benefits are judged to outweigh risks. Annex VII of the MDR empowers NBs to issue certificates of conformity with specific conditions, such as restrictions on the device's intended purpose or approved clinical indications, limits on the certificate's duration of validity, or requirements for specific Post-Market Clinical Follow-up (PMCF) studies (MDR Article 56.3; Annex IX) (1).

Although the MDR came into effect on 26 May 2021, the transition period between the MDD and full implementation of the MDR has been extended; for class III and class IIb implantable medical devices, the deadline to achieve compliance is now 31 December 2027 (16). Certain provisions of the MDR are not fully operational, such as the European Database on Medical Devices (EUDAMED) (17). In the future, EUDAMED will provide public access to all certificates of conformity, including any conditions or restrictions that have been applied. At present, since certificates issued under the MDD or transition period have not been publicly available, it is quite unclear how often notified bodies have exercised their authority to issue certificates with restrictions.

In order to understand current practices better, and to record challenges faced by NBs particularly during the implementation of the MDR, we conducted a survey of NBs to investigate how the medical device directives have been applied, focusing on the application of restrictions, limitations or requirements for PMCF on certificates. The objective was to provide insights as a baseline for comparison with future practices once the Medical Device Regulation (MDR) becomes fully operational.

## Policy options and implications

### Survey overview

The survey was developed within the Coordinating Research and Evidence for Medical Devices (CORE-MD) project (18), as part of a task concerning clinical evidence generation after market access and in particular, any restrictions or limitations imposed on certificates. It was addressed to NBs and implemented through the EU Survey tool (19). It was designed in close collaboration with experts from NBs, regulators, and the CORE-MD consortium. Initially conceived as a prospective study, the questionnaire evolved into a retrospective analysis due to operational challenges faced by NBs during the COVID-19 pandemic and the MDR transition period. Key objectives included determining the issuance, rejection, and application of restrictions or limitations on certificates, for class III and IIb implantable medical devices between August 2012 and May 2021 (Supplementary 1). Distribution commenced in March 2023 through multiple channels, including the European Association of Notified Bodies (Team-NB) and direct outreach via the list available on the NANDO website (New Approach Notified and Designated Organizations) (20). Potential participants were sent repeated invitations and offered various options to encourage their participation and collection of data – including online surveys, interviews, and virtual meetings.

The survey was distributed to 40 NBs; responses were received from 13 (Table 1). Of the 27 NBs which did not respond, 23 which had been invited by personal letters to participate, did not explain why they could not. Representatives from the other 4 NBs cited as reasons for their non-participation, that they were already overwhelmed with re-evaluating existing medical devices for compliance with the MDR, that they had too many other surveys to complete for the regulatory agencies, or that they lacked expertise in the subject matter.

**Table 1:** Results of the survey of European Notified Bodies: experience of applying conditions on certificates of conformity for high-risk medical devices.

NB	Numbers of certificates for class III and IIb implantable medical devices within the scope of the MDD & AIMDD^a^			Intended purpose, indications, area of medicine, and the type of restriction or limitation that was placed on the certificate		
	Total issued	Rejected	Issued with restrictions or limitations	Type of medical device	Intended purpose, indication or medical field	Nature of the restriction or limitation
1	305	35	0	N/A	N/A	N/A
2	50	2	0	N/A	N/A	N/A
3	1,000^b^	200	25	N/A	N/A	No details provided
4	14	0	0	N/A	N/A	N/A
5	47	0	0	N/A	N/A	N/A
6	N/A^c^	N/A^c^	0	N/A	N/A	N/A
7	34	2	0	N/A	N/A	N/A
8	0	0	0	N/A	N/A	N/A
9	30	8	0	N/A	N/A	N/A
10	118	5	0	N/A	N/A	N/A
11	441	75	4	Adhesion barrier	To reduce internal adhesions after surgery	Restriction of intended purpose
				Implantable suture	Wound healing	Restriction of intended purpose
				Dermal filler	Soft tissue augmentation	Restriction of intended purpose
				Surgical mesh	To provide support for weakened or damaged tissue	Restriction of intended purpose
12	563	0	0	N/A	N/A	N/A
13	N/A^c^	N/A^c^	3	Implantable glucose sensor (novel technology)	Diabetes mellitus	[Unknown effects of injections to prevent fibrosis around the sensor] Every implant to be enrolled into a registry. PMCF study to be performed. Manufacturer to report safety and performance every 3 months.
				Leadless pacemaker (novel technology)	Cardiology	[Lack of long-term data for expected lifetime of the device] Every implant to be enrolled into a registry. PMCF study to be performed. Manufacturer to report safety and performance every 3 months.
				Implantable brachytherapy seed to treat cancer (novel technology)	Oncology	[Unmet medical need] Every implant to be enrolled into a registry. PMCF study to be performed. Manufacturer to report safety and performance every 3 months.

NB Notified Body (note that the numbers refer only to their order in this table).

MDD Medical Device Directive; AIMD Active Medical Device Directive; N/A not applicable, or not available.

^a^ Time period: 01/08/2012–26/05/2021.

^b^ Some figures are informed approximations, when exact data were not available.

^c^ Notified bodies #6 and #13 reported that it was impossible for them to estimate the numbers of certificates awarded or refused (but #6 had issued no restrictions, while #13 had issued 3 certificates with conditions).

The survey estimated that 2,600 certificates had been granted under the MDD and AIMDD for Class III and IIb implantable medical devices, by the 11 participating NBs over the 8½ year survey period. Notably, one of the NBs provided an approximate count of 1,000 certificates that it had issued. Two NBs did not provide data on the total numbers of certificates or rejections that they had issued, but they stated that they had issued none with restrictions or limitations. Of the total of applications for certification retrieved from the survey responses, 327 had not been approved, equivalent to 11% of the estimated number of issued certificates. Three NBs issued certificates with restrictions (representing 1% of the total number). For one NB, the rate could not be estimated. Two NBs provided some additional data about 7 medical devices that had been issued with certificates with restrictions or limitations (see Table 1). 4 had a restriction on their intended purpose, and 3 required PMCF studies for new medical devices with uncertainty about their safety and performance.

It is worth mentioning that one NB provided us with detailed narratives describing the nature of the restrictions or limitations for 3 issued certificates. In these 3 cases, all appeared to offer a promising therapeutic option for an area that previously had an unmet medical need, but where the evidence was insufficient at launch. In each case, a PMCF study was required from the manufacturer, and subsequently those studies had informed a key follow-up decision. In one case, that follow-up decision led to the certificate being cancelled, in one case the device could continue with no further restrictions, and in one case the PMCF was continued. These examples show that that although the MDR does not employ the term “conditional certification” in the same way as the FDA, NBs can issue certificates with a requirement for specific PMCF and that this can be an effective and valuable tool to balance the need for patients' access to novel technologies with the need to guarantee that those technologies are safe and effective.

## Key findings

### Functioning of the EU regulatory system

• Conditional certifications are permitted by the MDR but underutilized in practice: In general, conditional certifications were applied infrequently by NBs. These certifications were mostly limited in scope, typically restricting the intended purpose or mandating post-market surveillance to address unresolved safety concerns of novel technology. Instead, NBs may be rejecting applications for certification where the evidence base is weak or uncertain, potentially limiting access within the EU to innovative and orphan devices.
• Variation between NBs in their practices: There were very large differences between NBs in placing conditions on certificates of conformity for high-risk medical devices. This variability influences whether and how NBs restrict or limit their use until more clinical data becomes available.

### Challenges faced by Notified Bodies

• Limited availability of data and tracking: NBs generally keep track of their certificates, however, they may not routinely track the specific details related to restrictions or limitations within the certificates. This makes it difficult to gather comprehensive data, making it difficult for policy makers to combine data from all the NBs to understand the full picture and improve the processes and policies that lead to the issuance of certificates with restrictions or limitations.
• Workload of NBs: A significant number of Notified Bodies did not participate in the survey, citing reasons such as workload due to the MDD/MDR transition period and the overall number of requests for information.

## Actionable recommendations

**Figure 1:**
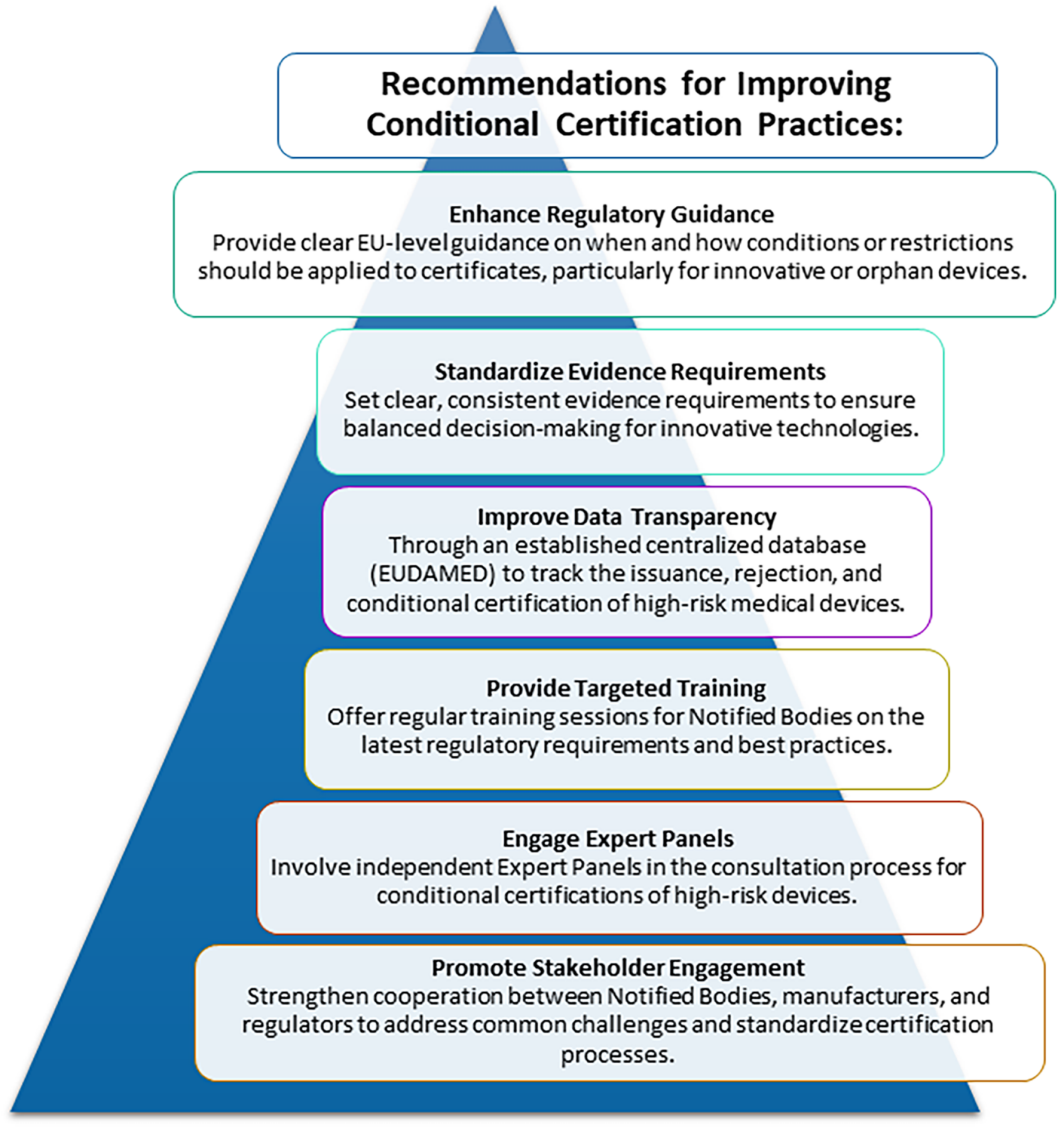
Recommendations to address challenges faced by Notified Bodies and affecting functioning of EU regulatory system in applying conditions and restrictions to certificates for high-risk medical devices.

There is an expected announced revision/public consultation of the MDR within the next months (21). The submission from the Biomedical Alliance in Europe will include proposals from the CORE-MD project, including the recommendation to increase the use of conditions on certificates (Figure 1).

• Regulatory guidance:

Regulatory authorities and the European Commission should provide more detailed recommendations, such as the EU guidance on orphan devices (MDCG 2024-10) (22), on the restrictions or limitations that can be applied to certificates issued, and on the circumstances when they might (or might not) be appropriate. Examples would include promising technologies with identifiable gaps in their evidence, for populations with an unmet need or new methodological approaches regarding the trial design. A feasible and high-quality PMCF study proposed by the manufacturer could address the evidence gaps, if agreed at the time of launch in a protocol between the manufacturer and the NB, and if credible sanctions are applied reliably if the PMCF study is not completed (e.g., cancellation of the certificate). This would help to standardize practices and ensure consistent application across different NBs.

• Standardization of evidence requirements:

With the transition from the directives to the regulation, the implementation of rules has become binding and uniform across all Member States, replacing some previous flexibility that allowed national variations. However, the review and approval of applications by manufacturers for their devices are undertaken in Europe by notified bodies (not national regulatory agencies) and that did not change with the transition to the EU MDR*.* Without a clear conditional access pathway, there is a risk of inconsistent application of limitations and restrictions. Without such a mechanism, Europe risks making definitive (negative) regulatory decisions in cases where more flexible, conditional approaches could better balance innovation and safety, potentially limiting patient access to promising new technologies.

• Enhanced data transparency:

Completing the establishment of a centralized database for tracking the issuance, rejection, and conditional certification of medical devices, that will be facilitated through EUDAMED (20), would represent a critical step towards improving transparency and facilitating better decision-making. The European medical device regulatory system would greatly benefit from adopting transparency levels similar to those in the pharmaceutical sector or other jurisdictions. For example, information is readily accessible on conditional marketing authorizations for medicines in Europe, and by the US FDA and the Chinese NPMA, both for medicines and medical devices, in their annual reports (23–25). In contrast, obtaining similar data for medical devices in Europe has proved to be challenging. Team-NB publishes annual reports (26) but they lack detailed information, and we could not acquire this data from our survey either.

• Training and support:

NBs should use their regular training and updates on the latest regulatory requirements to coordinate best practices in certification. Capacity building initiatives of this sort have been funded by the European Commission in other fields (such as the tender launched to build capacity for the implementation of the new HTA regulation) (27). Similar capacity-building initiatives in the field of medical devices would ensure that all NBs are well-equipped to apply conditions effectively and uniformly.

• Involvement of Expert Panels:

The MDR required the establishment of independent Expert Panels to advise the EU Commission, the Medical Device Coordination Group, Member States, NBs and manufacturers on the scientific assessment of clinical evidence provided for certain high-risk medical devices and *in vitro* diagnostic medical devices (Articles 106, 48 and 54) (28). The Expert Panels could also be assigned a role to determine whether high-risk devices should qualify for special pathways with conditional approval.

• Stakeholder engagement:

NBs are not allowed to engage in consultation with manufacturers about their evidence development plans, as this could be perceived as a conflict of interest. Nevertheless, at some level collaboration between NBs, manufacturers, and regulatory bodies is essential to address common challenges and share best regulatory practices. The absence of an early consultation system with NBs in Europe has resulted in suboptimal regulatory outcomes, along with increased costs and delays in conformity assessments (29). Such a collaboration is being currently explored in the framework of the Joint Scientific Consultation subgroup of HTA under the new Health Technology Assessment Regulation (HTAR) (30); as an interim arrangement, a parallel approach is offered for consultation and scientific advice from the EMA and from the HTA body.

That gives the opportunity to manufacturers to seek advice on evidence requirements for both regulators and HTA to promote optimal and robust evidence generation (31). A similar collaboration could streamline the development of evidence for special high-risk medical devices, clarifying regulatory expectations and providing a clearer pathway to market entry. It might be associated with more administrative complexity, and could provide conflicting advice, but such challenges could be addressed satisfactorily if more regulatory resources were allocated to ensuring a functioning and coordinated system for issuing certificates with conditions.

## Conclusions

Despite the limited number of responses, our survey shed light on the current landscape of conditional certification practices for high-risk medical devices in the EU. Although a potentially useful regulatory tool to provide access to innovative and orphan devices, NBs rarely apply restrictions, limitations and/or requirements for PMCF on certificates for high-risk medical devices. The substantial variability in responses between NBs and the limited availability of granular information on issued certificates, rejections, restrictions or limitations, makes it challenging to assess the effectiveness and uniformity of these practices. It would be beneficial to replicate this study once the MDR is fully implemented, to analyze whether stricter requirements imposed by the new regulation have influenced the issuance of certificates of conformity with specific conditions and their subsequent monitoring. National regulatory authorities and the European Commission should provide clearer guidance to promote the use of certificates with conditions, where appropriate, and to improve consistency between NBs, which could in consequence facilitate and streamline NBs workflow. Access to medical technologies in situations of urgent need can be balanced by conditions to improve safety for patients, while creating an environment that is more favorable to innovation. Issuing more certificates with conditions, however, implies accepting the possibility of increased risk from devices with limited evidence, and could add to the workload of NBs because of the need for more intensive post- launch evidence generation.
