# Supplementary figures and images for: Survey of Notified Bodies reveals very limited use of conditional certification for high-risk medical devices
Source: Front Med Technol. 2025 Feb 3;7:1504294. doi: 10.3389/fmedt.2025.1504294 (PMC11830701; doi:10.3389/fmedt.2025.1504294)

Supplementary Material

# Survey Form of Notified Bodies experiences


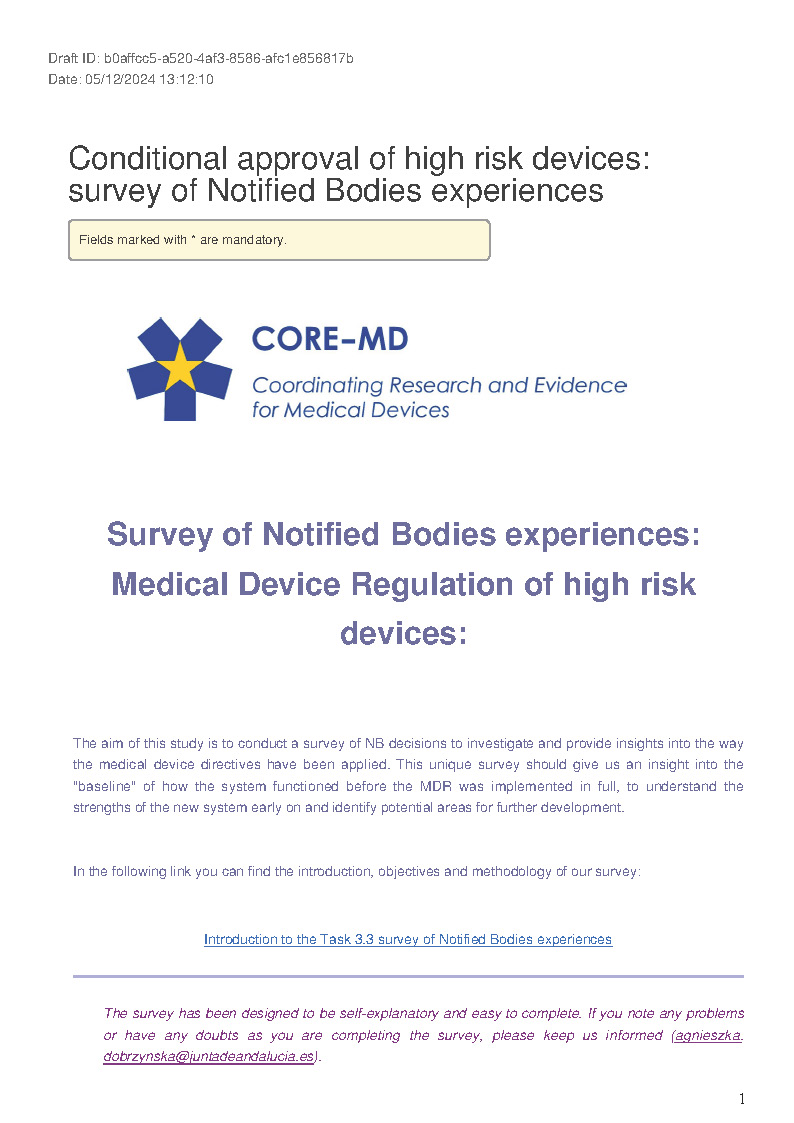


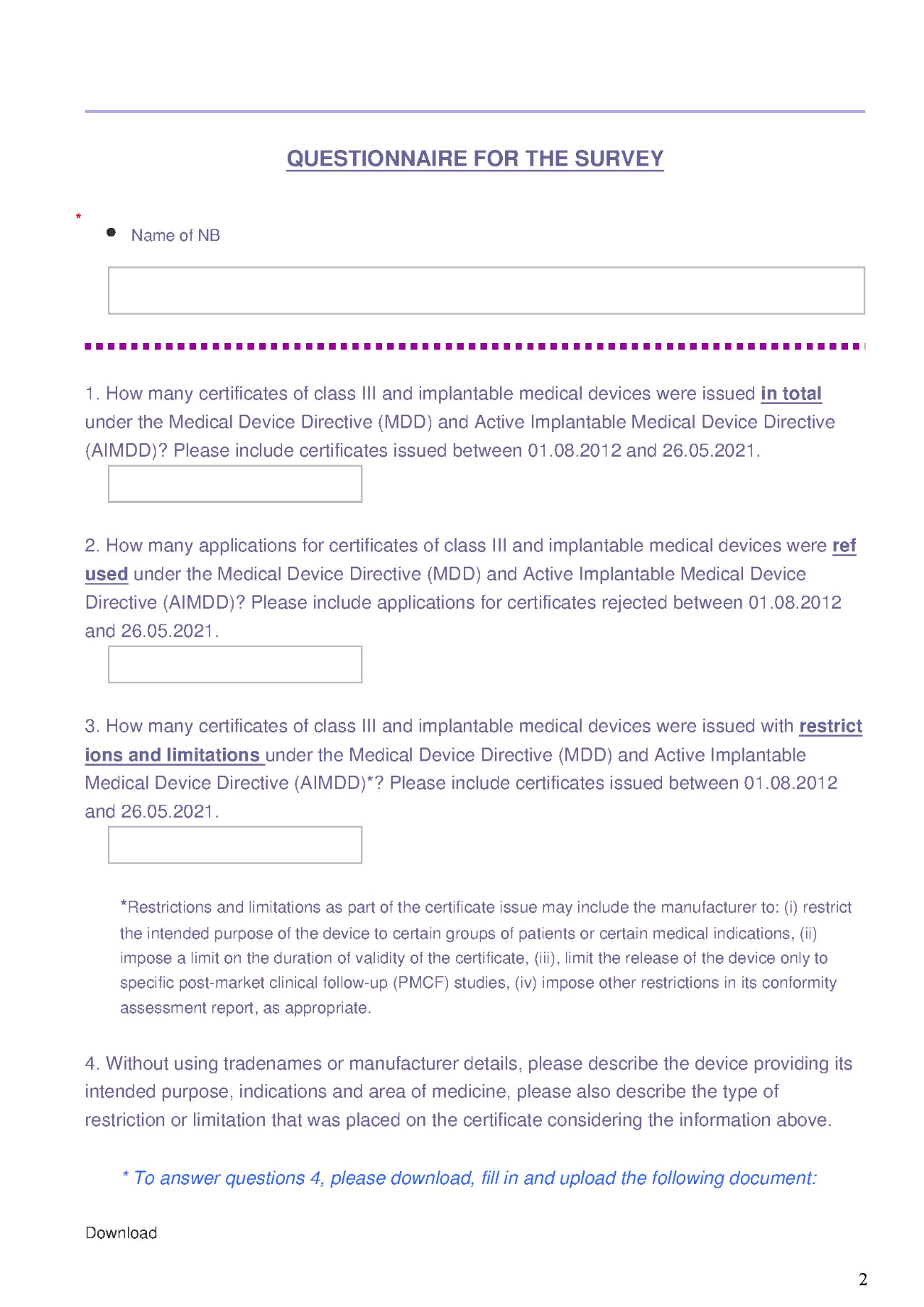


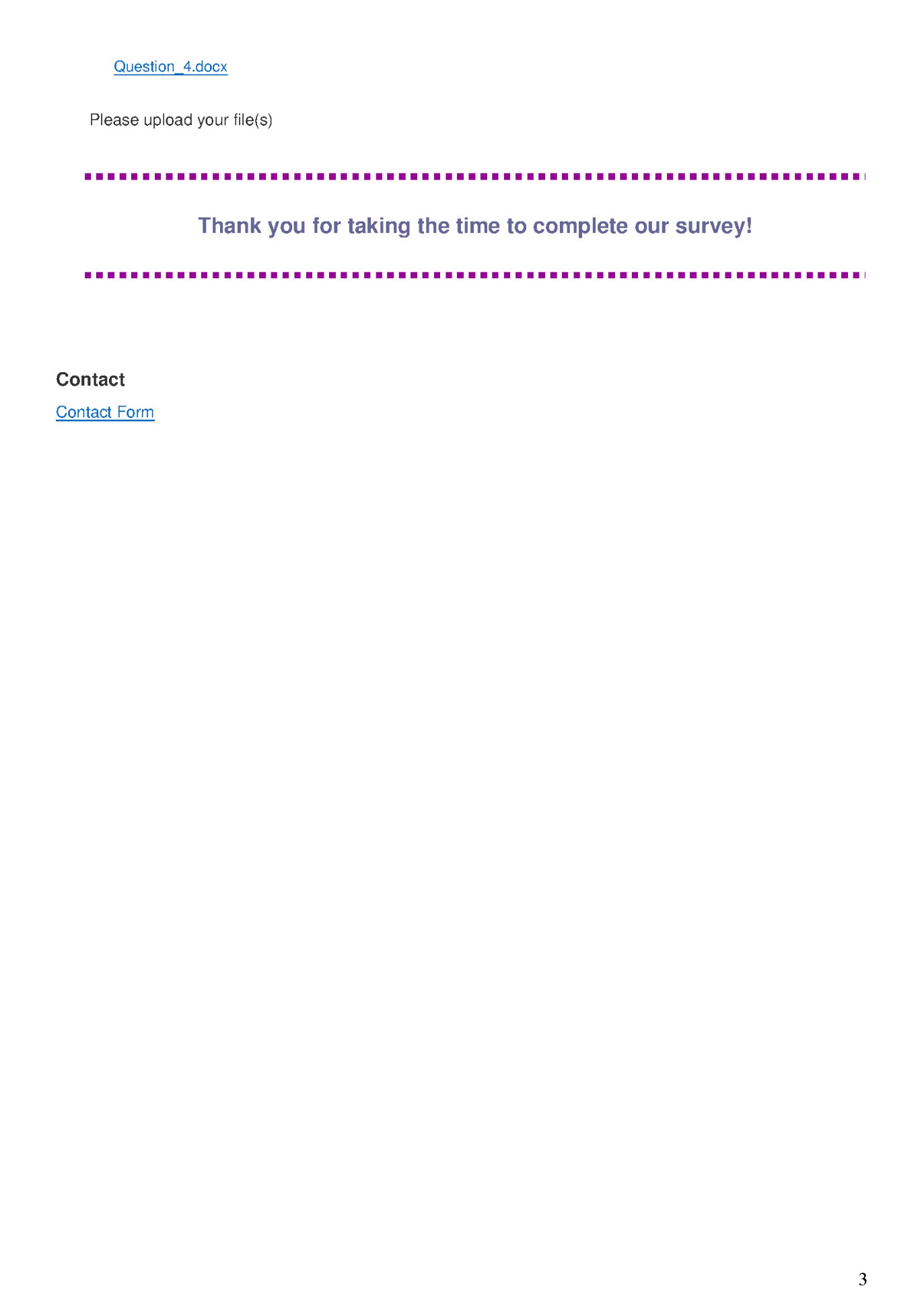

Supplement: Supplementary file 1 [file Datasheet1.docx]
